# Ten Years of Pathway Analysis: Current Approaches and Outstanding Challenges

**DOI:** 10.1371/journal.pcbi.1002375

**Published:** 2012-02-23

**Authors:** Purvesh Khatri, Marina Sirota, Atul J. Butte

**Affiliations:** 1 Division of Systems Medicine, Department of Pediatrics, Stanford University School of Medicine, Stanford, California, United States of America; 2 Lucile Packard Children's Hospital, Palo Alto, California, United States of America; The Centre for Research and Technology, Hellas, Greece

## Abstract

Pathway analysis has become the first choice for gaining insight into the underlying biology of differentially expressed genes and proteins, as it reduces complexity and has increased explanatory power. We discuss the evolution of knowledge base–driven pathway analysis over its first decade, distinctly divided into three generations. We also discuss the limitations that are specific to each generation, and how they are addressed by successive generations of methods. We identify a number of annotation challenges that must be addressed to enable development of the next generation of pathway analysis methods. Furthermore, we identify a number of methodological challenges that the next generation of methods must tackle to take advantage of the technological advances in genomics and proteomics in order to improve specificity, sensitivity, and relevance of pathway analysis.

## Introduction

Techniques such as high-throughput sequencing and gene/protein profiling techniques have transformed biological research by enabling comprehensive monitoring of a biological system. Irrespective of the technology used, analysis of high-throughput data typically yields a list of differentially expressed genes or proteins. This list is extremely useful in identifying genes that may have roles in a given phenomenon or phenotype. However, for many investigators, this list often fails to provide mechanistic insights into the underlying biology of the condition being studied. In this way, the advent of high-throughput profiling technologies presents a new challenge, that of extracting meaning from a long list of differentially expressed genes and proteins.

One approach to this challenge has been to simplify analysis by grouping long lists of individual genes into smaller sets of related genes or proteins. This approach reduces the complexity of analysis. Researchers have developed a large number of knowledge bases to help with this task. The knowledge bases describe biological processes, components, or structures in which individual genes and proteins are known to be involved in, as well as how and where gene products interact with each other. One example of this idea is to identify groups of genes that function in the same pathways.

Analyzing high-throughput molecular measurements at the functional level is very appealing for two reasons. First, grouping thousands of genes, proteins, and/or other biological molecules by the pathways they are involved in reduces the complexity to just several hundred pathways for the experiment. Second, identifying active pathways that differ between two conditions can have more explanatory power than a simple list of different genes or proteins [1].

The goals of this review are to i) describe the existing knowledge base–driven pathway analysis methods, ii) discuss limitations of each class of methods, and iii) describe the challenges not yet addressed by any method.

## Existing Pathway Analytic Approaches

The term “pathway analysis” has been used in very broad contexts in the literature [2]. It has been applied to the analysis of Gene Ontology (GO) terms (also referred to as a “gene set”), physical interaction networks (e.g., protein–protein interactions), kinetic simulation of pathways, steady-state pathway analysis (e.g., flux-balance analysis), and in the inference of pathways from expression and sequence data. However, the definition of a “pathway” in some of these uses may be misleading or incorrect. For instance, the cellular compartment ontology in GO does not describe a pathway.

It is beyond the scope of this review to discuss the large number of analytic methods covered by such a broad application of the term “pathway analysis.” Therefore, this review focuses on methods that exploit pathway knowledge in public repositories such as GO or Kyoto Encyclopedia of Genes and Genomes (KEGG), rather than on methods that infer pathways from molecular measurements. We call this approach *knowledge base–driven* pathway analysis. It identifies pathways that may be affected in a condition by correlating information in at least one pathway knowledge base with gene expression patterns for the condition. The result is differential expression of a set of genes or proteins rather than a list of individual genes.

Instead of individually reviewing a large number of pathway analysis approaches, our goal here is to group approaches by the type of analysis they perform and discuss their relative merits. However, for those desiring specific information about individual tools, Text S2 provides feature comparisons for a number of individual tools in each group.

Virtually all of the approaches and tools discussed here are independent of the data generated from most high-throughput technologies, including next-generation sequencing data and the knowledge bases used for pathway annotations. In this review, we use gene expression measurements as example data for discussing and explaining various approaches.

### First Generation: Over-Representation Analysis (ORA) Approaches

**Figure 1:**
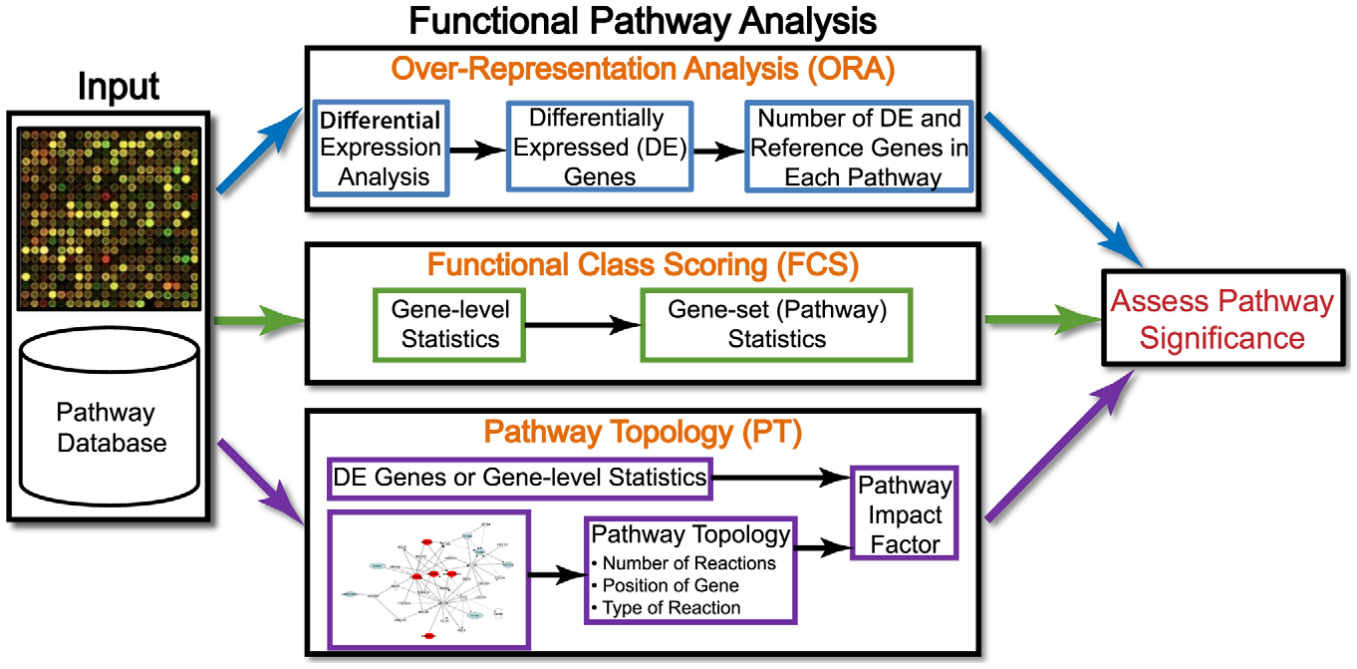
Overview of existing pathway analysis methods using gene expression data as an example. Note that this overview is equally applicable to molecular measurements using proteomics, and any other high-throughput technologies. The data generated by an experiment using a high-throughput technology (e.g., microarray, proteomics, metabolomics), along with functional annotations (pathway database) of the corresponding genome, are input to virtually all pathway analysis methods. While ORA methods require that the input is a list of differentially expressed genes, FCS methods use the entire data matrix as input. In addition to functional annotations of a genome, PT-based methods utilize the number and type of interactions between gene products, which may or may not be a part of a pathway database. The result of every pathway analysis method is a list of significant pathways in the condition under study. DE, differentially expressed.

The immediate need for functional analysis of microarray gene expression data and the emergence of GO during that period gave rise to over-representation analysis (ORA), which statistically evaluates the fraction of genes in a particular pathway found among the set of genes showing changes in expression (Table 1). It is also referred to as “2×2 table method” in the literature [3]. ORA uses one or more variations of the following strategy [4]–[11] (Figure 1): first, an input list is created using a certain threshold or criteria. For example, a researcher may choose genes that are differentially over- or under-expressed in a given condition at a false discovery rate (FDR) of 5%. Then, for each pathway, input genes that are part of the pathway are counted. This process is repeated for an appropriate background list of genes (e.g., all genes measured on a microarray). Next, every pathway is tested for over- or under-representation in the list of input genes. The most commonly used tests are based on the hypergeometric, chi-square, or binomial distribution. We refer the readers to recent comparisons of ORA tools for more details [12], [13]. Many of the ORA tools differ very slightly from each other as they use the same statistical tests as well as overlapping pathway databases (Table S1).

**Table 1:** Examples of pathway analysis tools in each generation.

Name	Availability	Reference
**ORA tools**		
Onto-Express	Web (http://vortex.cs.wayne.edu)	[4], [5]
GenMAPP	Standalone (http://www.genmapp.org)	[11], [71]
GoMiner	Standalone, Web (http://discover.nci.nih.gov/gominer)	[72], [73]
FatiGO	Web (http://babelomics.bioinfo.cipf.es)	[74]
GOstat	Web (http://gostat.wehi.edu.au)	[7]
FuncAssociate	Web (http://llama.mshri.on.ca/funcassociate/)	[6]
GOToolBox	Web (http://genome.crg.es/GOToolBox/)	[10]
GeneMerge	Standalone, Web (http://genemerge.cbcb.umd.edu/)	[9]
GOEAST	Web (http://omicslab.genetics.ac.cn/GOEAST/)	[75]
ClueGO	Standalone (http://www.ici.upmc.fr/cluego/)	[76]
FunSpec	Web (http://funspec.med.utoronto.ca/)	[77]
GARBAN	Web	[78]
GO:TermFinder	Standalone (http://search.cpan.org/dist/GO-TermFinder/)	[8]
WebGestalt	Web (http://bioinfo.vanderbilt.edu/webgestalt/)	[79]
agriGO	Web (http://bioinfo.cau.edu.cn/agriGO/)	[80]
GOFFA	Standalone, Web (http://edkb.fda.gov/webstart/arraytrack/)	[81]
WEGO	Web (http://wego.genomics.org.cn/cgi-bin/wego/index.pl)	[82]
**FCS tools**		
GSEA	Standalone (http://www.broadinstitute.org/gsea/)	[21], [29]
sigPathway	Standalone (BioConductor)	[22]
Category	Standalone (BioConductor)	[24]
SAFE	Standalone (BioConductor)	[30]
GlobalTest	Standalone (BioConductor)	[15]
PCOT2	Standalone (BioConductor)	[17]
SAM-GS	Standalone (http://www.ualberta.ca/~yyasui/software.html)	[83]
Catmap	Standalone (http://bioinfo.thep.lu.se/catmap.html)	[84]
T-profiler	Web (http://www.t-profiler.org)	[85]
FunCluster	Standalone (http://corneliu.henegar.info/FunCluster.htm)	[86]
GeneTrail	Web (http://genetrail.bioinf.uni-sb.de)	[87]
GAzer	Web	[88]
**PT-based tools**		
ScorePAGE	No implementation available	[37]
Pathway-Express	Web (http://vortex.cs.wayne.edu)	[38], [39]
SPIA	Standalone (BioConductor)	[40]
NetGSA	No implementation available	[43]

#### Limitations

Despite the availability of a large number of tools and their widespread usage, ORA has a number of limitations. First, the different statistics used by ORA (e.g., hypergeometric distribution, binomial distribution, chi-square distribution, etc.) are independent of the measured changes. This means that these tests consider the number of genes alone and ignore any values associated with them such as probe intensities. By discarding this data, ORA treats each gene equally. However, the information about the extent of regulation (e.g., fold-changes, significance of a change, etc.) can be useful in assigning different weights to input genes, as well as to the pathways they are involved in, which in turn can provide more information than current ORA approaches.

Second, ORA typically uses only the most significant genes and discards the others. For instance, the input list of genes from a microarray experiment is usually obtained using an arbitrary threshold (e.g., genes with fold-change 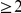 and/or *p*-values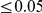). With this method, marginally less significant genes (e.g., fold-change = 1.999 or *p*-value = 0.051) are missed, resulting in information loss. Breitling et al. addressed this problem by proposing an ORA method for avoiding thresholds. It uses an iterative approach that adds one gene at a time to find a set of genes for which a pathway is most significant [14].

Third, by treating each gene equally, ORA assumes that each gene is independent of the other genes. However, biology is a complex web of interactions between gene products that constitute different pathways. One goal of gene expression analysis might be to gain insights into *how* interactions between gene products are manifested as changes in gene expression. A strategy that assumes the genes are independent is significantly limited in its ability to provide insights in this regard. Furthermore, assuming independence between genes amounts to “competitive null hypothesis” testing (see below), which ignores the correlation structure between genes. Consequently, the estimated significance of a pathway may be biased or incorrect.

Fourth, ORA assumes that each pathway is independent of other pathways, which is erroneous. For instance, GO defines a biological process as a series of events accomplished by one or more *ordered* assemblies of molecular functions (http://www.geneontology.org/GO.doc.shtml). Another example of dependence between pathways is the cell cycle pathway in KEGG (http://www.genome.jp/kegg/pathway/hsa/hsa04110.html), where the presence of a growth factor activates the MAPK signaling pathway. This, in turn, activates the cell cycle pathway. No ORA methods account for this dependence between molecular functions in GO and signaling pathways in KEGG.

### Second Generation: Functional Class Scoring (FCS) Approaches

The hypothesis of functional class scoring (FCS) is that although large changes in individual genes can have significant effects on pathways, weaker but coordinated changes in sets of functionally related genes (i.e., pathways) can also have significant effects. With few exceptions [15]–[17], all FCS methods use a variation of a general framework that consists of the following three steps [18] (Figure 1; Table 1): first, a gene-level statistic is computed using the molecular measurements from an experiment. This involves computing differential expression of individual genes or proteins. Statistics currently used at gene-level include correlation of molecular measurements with phenotype [19], ANOVA [20], Q-statistic [15], signal-to-noise ratio [21], *t*-test [20], [22], and Z-score [23]. Although the choice of a gene-level statistic has a negligible effect on the identification of significantly enriched gene sets [18], when there are few biological replicates, a regularized statistic may be better. Furthermore, untransformed gene-level statistics can fail to identify pathways with up- and down-regulated genes. In this case, transformation of gene-level statistics (e.g., absolute values, squared values, ranks, etc.) is preferable [18], [24].

Second, the gene-level statistics for all genes in a pathway are aggregated into a single pathway-level statistic. This statistic can be multivariate [17], [25]–[28] and account for interdependencies among genes, or it can be univariate [22], [24] and disregard interdependencies among genes. The pathway-level statistics used by current approaches include the Kolmogorov-Smirnov statistic [21], [29], sum, mean, or median of gene-level statistic [24], the Wilcoxon rank sum [30], and the maxmean statistic [31]. Irrespective of its type, the power of a pathway-level statistic can depend on the proportion of differentially expressed genes in a pathway, the size of the pathway, and the amount of correlation between genes in the pathway. Interestingly, although multivariate statistics are expected to have higher statistical power, univariate statistics show more power at stringent cutoffs when applied to real biological data (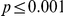), and equal power as multivariate statistics at less stringent cutoffs (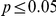) [1].

The final step in FCS is assessing the statistical significance of the pathway-level statistic. When computing statistical significance, the null hypothesis tested by current pathway analysis approaches can be broadly divided into two categories: i) competitive null hypothesis and ii) self-contained null hypothesis [3], [18], [22], [31]. A self-contained null hypothesis permutes class labels (i.e., phenotypes) for each sample and compares the set of genes in a given pathway with itself, while ignoring the genes that are not in the pathway. On the other hand, a competitive null hypothesis permutes gene labels for each pathway, and compares the set of genes in the pathway with a set of genes that are not in the pathway. Text S2 has a detailed discussion on the differences between the two null hypotheses.

FCS methods address three limitations of ORA. First, they do not require an arbitrary threshold for dividing expression data into significant and non-significant pools. Rather, FCS methods use all available molecular measurements for pathway analysis. Second, while ORA completely ignores molecular measurements when identifying significant pathways, FCS methods use this information in order to detect coordinated changes in the expression of genes in the same pathway. Finally, by considering the coordinated changes in gene expression, FCS methods account for dependence between genes in a pathway, which ORA does not.

#### Limitations

Although FCS is an improvement over ORA [19], [22], it also has several limitations. First, similar to ORA, FCS analyzes each pathway independently. This is a limitation because a gene can function in more than one pathway, meaning that pathways can cross and overlap. Consequently, in an experiment, while one pathway may be affected in an experiment, one may observe other pathways being significantly affected due to the set of overlapping genes. Such a phenomenon is very common when using the GO terms to define pathways due to the hierarchical nature of the GO.

Second, many FCS methods use changes in gene expression to rank genes in a given pathway, and discard the changes from further analysis. For instance, assume that two genes in a pathway, A and B, are changing by 2-fold and 20-fold, respectively. As long as they both have the same respective ranks in comparison with other genes in the pathway, most FCS methods will treat them equally, although the gene with the higher fold-change should probably get more weight. Importantly, however, considering only the ranks of genes is also advantageous, as it is more robust to outliers. A notable exception to this scenario is approaches that use gene-level statistics (e.g., t-statistic) to compute pathway-level scores. For example, an FCS method that computes a pathway-level statistic as a sum or mean of the gene-level statistic accounts for a relative difference in measurements (e.g., Category, SAFE in Table S2).

### Third Generation: Pathway Topology (PT)-Based Approaches

A large number of publicly available pathway knowledge bases provide information beyond simple lists of genes for each pathway. Unlike GO and the Molecular Signatures Database (MSigDB), these knowledge bases also provide information about gene products that interact with each other in a given pathway, how they interact (e.g., activation, inhibition, etc.), and where they interact (e.g., cytoplasm, nucleus, etc.). These knowledge bases include KEGG [32], MetaCyc [33], Reactome [34], RegulonDB [35], STKE (http://stke.sciencemag.org), BioCarta (http://www.biocarta.com), and PantherDB [36].

ORA and FCS methods consider only the number of genes in a pathway or gene coexpression to identify significant pathways, and ignore the additional information available from these knowledge bases. Hence, even if the pathways are completely redrawn with new links between the genes, as long as they contain the same set of genes, ORA and FCS will produce the same results. Pathway topology (PT)-based methods (Table 1; Table S3) have been developed to utilize the additional information. PT-based methods are essentially the same as FCS methods in that they perform the same three steps as FCS methods. The key difference between the two is the use of pathway topology to compute gene-level statistics.

Rahnenfuhrer et al. proposed ScorePAGE, which computes similarity between each pair of genes in a pathway (e.g., correlation, covariance, etc.) [37]. The similarity measurement between each pair of genes is analogous to gene-level statistics in FCS methods, which is averaged to compute a pathway-level score. However, instead of giving equal weight to all pairwise similarities, ScorePAGE divides the pairwise similarities by the number of reactions needed to connect two genes in a given pathway (Figure 1). Although the approach is designed to analyze metabolic pathways, it is theoretically also applicable to signaling pathways.

A recent impact factor (IF) analytic approach was proposed to analyze signaling pathways. IF considers the structure and dynamics of an entire pathway by incorporating a number of important biological factors, including changes in gene expression, types of interactions, and the positions of genes in a pathway [38], [39] (Figure 1). Briefly, IF analysis models a signaling pathway as a graph, where nodes represent genes and edges represent interactions between them. Further, it defines a gene-level statistic, called perturbation factor (PF) of a gene, as a sum of its measured change in expression and a linear function of the perturbation factors of all genes in a pathway (see Equation 1 in Text S1). Because the PF of each gene is defined by a linear equation, the entire pathway is defined as a linear system. Representing a pathway as a linear system also addresses loops in the pathways [39]. The IF of a pathway (pathway-level statistic) is defined as a sum of PF of all genes in a pathway (see Equation 2 in Text S1). IF analysis was recently improved to address the dominating effect of change in expression on PF and high false positive rate for a small list of input genes [40].

FCS methods that use correlations among genes [19], [41] implicitly assume that the underlying network, as defined by the correlation structure, does not change as the experimental conditions change. However, this assumption may be inaccurate. For example, the correlation structure between *ARG2* and other genes in the urea-cycle pathway changes with a change in expression of *ARG2* [42], suggesting changes in the topology of the pathway.

Shojaie et al. proposed a method, called NetGSA, that accounts for the the change in correlation as well as the change in network structure as experimental conditions change [43]. Their approach, like IF analysis, models gene expression as a linear function of other genes in the network. However, it differs from IF in two aspects. First, it accounts for a gene's baseline expression by representing it as a latent variable in the model. Second, it requires that the pathways be represented as directed acyclic graphs (DAGs). If a pathway contains cycles, NetGSA requires additional latent variables affecting the nodes in the cycle. In contrast, IF analysis does not impose any constraint on the structure of a pathway [39].

#### Limitations

Although PT-based methods are difficult to generalize, they have several common limitations. One obvious problem is that true pathway topology is dependent on the type of cell due to cell-specific gene expression profiles and condition being studied. However, this information is rarely available and is fragmented in knowledge bases, even if it is fully understood [44]. As annotations improve, these approaches are expected to become more useful. Other limitations of PT-based methods include the inability to model dynamic states of a system and the inability to consider interactions between pathways due to weak inter-pathway links to account for interdependence between pathways. These limitations are discussed in detail in the Outstanding Challenges section below.

## Outstanding Challenges in Pathway Analysis

The current challenges in pathway analysis can be divided into two broad categories: i) annotation challenges and ii) methodological challenges. We believe that development of the next generation of pathway analytic approaches will require improvement of the existing annotations. It is necessary to create accurate, high resolution knowledge bases with detailed condition-, tissue-, and cell-specific functions of each gene. These knowledge bases will allow investigators to model an organism's biology as a dynamic system, and will help predict changes in the system due to factors such as mutations or environmental changes.

### Annotation Challenges

#### Low resolution knowledge bases

**Figure 2:**
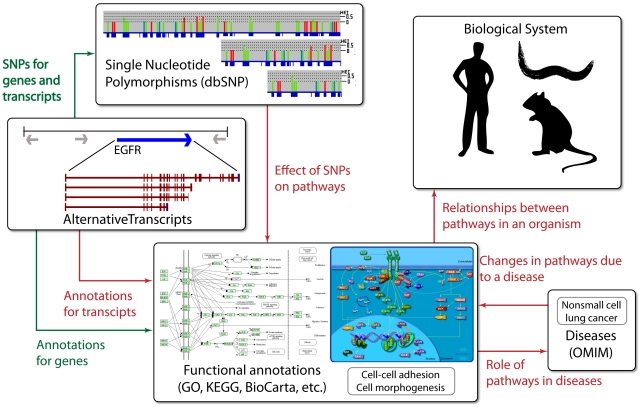
Overview of low resolution, missing, and incomplete information. Green arrows represent abundantly available information, and red arrows represent missing and/or incomplete information. The ultimate goal of pathway analysis is to analyze a biological system as a large, single network. However, the links between smaller individual pathways are not yet well known. Furthermore, the effects of a SNP on a given pathway are also missing from current knowledge bases. While some pathways are known to be related to a few diseases, it is not clear whether the changes in pathways are the cause for those diseases or the downstream effects of the diseases.

Recent technological advances in genomics and proteomics are generating data at unprecedented high resolution. As a result, there is a need for correspondingly high resolution annotation knowledge bases. For instance, using RNA-seq, more than 90% of the human genome is estimated to be alternatively spliced. Multiple transcripts from the same gene may have related, distinct, or even opposing functions [45]. Similarly, genome-wide association studies (GWASs) have identified a large number of SNPs that may be involved in different conditions and diseases. However, current knowledge bases only specify which genes are active in a given pathway. It is essential that they also begin specifying other information, such as *transcripts* that are active in a given pathway or how a given SNP affects a pathway (Figure 2). To the best of our knowledge, because of these low resolution knowledge bases, every available pathway analysis tool first maps the input to a non-redundant namespace, typically an Entrez Gene ID [46]. Arguably, this type of mapping is advantageous [47], although it can be non-trivial [48] and dynamic [49], as it allows the existing pathway analysis approaches to be independent of the technology used in the experiment. However, mapping in this way also results in the loss of important information that may have been provided because a specific technology was used. For instance, *XRN2a*, a variant of gene *XRN2*, is expressed in several human tissues, whereas another variant of the same gene, *XRN2b*, is mainly expressed in blood leukocytes [50]. Although RNA-seq can quantify expression of both variants, mapping both transcripts to a single gene causes loss of tissue-specific information, and possibly even condition-specific information.

Therefore, before pathway analysis can exploit current and future technological advances in biotechnology, it is critically important to annotate exact transcripts and SNPs that participate in a given pathway. While new approaches are being developed in this regard, they may not yet be adequate. For example, Braun et al. proposed a method for analyzing SNP data from a GWAS [51]. However, this approach still relies on mapping multiple SNPs to a single gene, followed by gene-to-pathway mapping [51]. Hence, the limited applicability of today's knowledge bases to emerging technologies shows the need for increased resolution of knowledge bases.

#### Incomplete and inaccurate annotations

**Figure 3:**
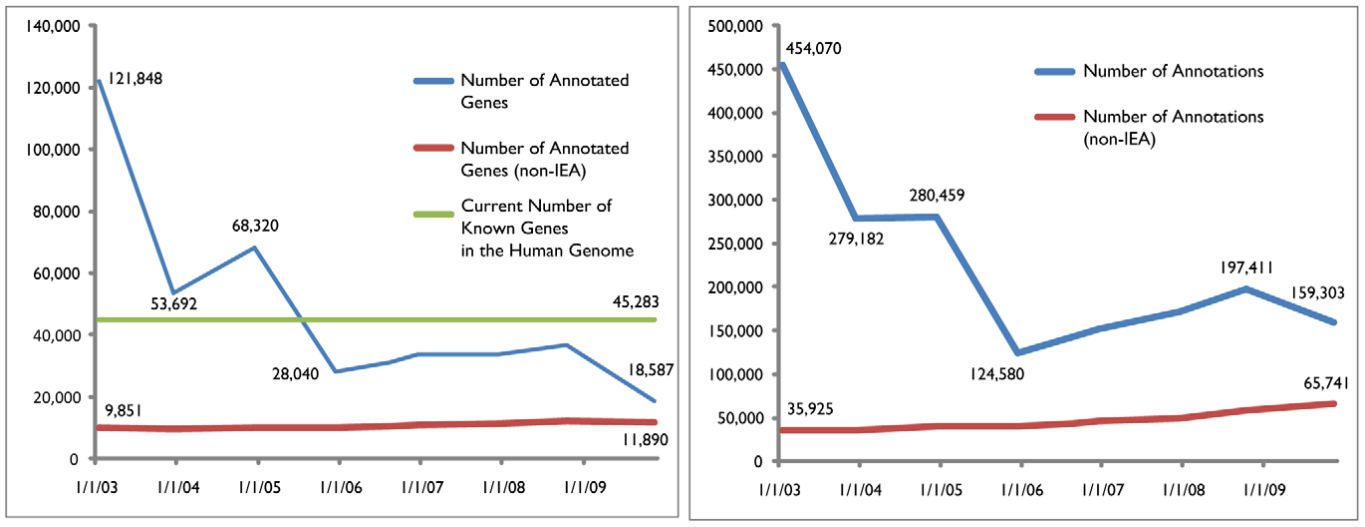
Number of GO-annotated genes (left panel) and number of GO annotations (right panel) for human from January 2003 to November 2009. As the estimated number of known genes in the human genome is adjusted (between January 2003 and December 2003) and annotation practices are modified (between December 2004 and December 2005, and between October 2008 and November 2009), one can argue that, although the number of annotated genes and the annotations are decreasing (which is mainly due to the adjusted number of genes in the human genome and changes in the annotation process), the quality of annotations is improving, as demonstrated by the steady increase in non-IEA annotations and the number of genes with non-IEA annotations. However, the increase in the number of genes with non-IEA annotations is very slow. In almost 7 years, between January 2003 and November 2009, only 2,039 new genes received non-IEA annotations. At the same time, the number of non-IEA annotations increased from 35,925 to 65,741, indicating a strong research bias for a small number of genes.

Despite the enormous number of annotations available in the public domain, a surprisingly large number of genes are still not annotated. For instance, the November 2009 release of GO contained entries for 18,587 human genes annotated with at least one GO term (Figure 3). Many of the genes are hypothetical, predicted, or pseudogenes. For example, although the number of protein-coding genes in the human genome is estimated to be between 20,000 and 25,000 [52], according to National Center for Biotechnology Information (NCBI) Entrez Gene, there are 45,283 human genes, of which 14,162 are pseudogenes (Table S4). One could argue that the pseudogenes should not be included when evaluating functional annotation coverage. However, pseudogene-derived small interfering RNAs have been shown to regulate gene expression in mouse oocytes [53]. Furthermore, GO provides annotations for 271 pseudogenes. A widely used DNA microarray, Affymetrix HG U133 plus 2.0, contains 1,026 probe sets that correspond to 823 pseudogenes. Based on these examples, we believe that the pseudogenes should be included in the count when estimating annotation coverage for the human genome.

In addition to incomplete annotations, many of the existing annotations are of low quality and may be inaccurate. For instance, >95% of the annotations in the October 2007 release of GO had the evidence code “inferred from electronic annotations (IEA)”. These annotations are the only ones in GO that are not curated manually [54]. Annotations inferred from indirect evidence are considered to be of lower quality than those derived from direct experimental evidence, although this opinion has not been shown robustly [54]. If the annotations with IEA code are removed, the number of genes with good quality annotations in the November 2009 release of human GO annotations is reduced from 18,587 to 11,890 (Figure 3).

It is very likely that the reduced number of annotations and annotated genes since January 2003 is an indicator of improving quality. This is due in part to the fact that the number of genes in a genome are continuously being adjusted and the functional annotation algorithms are being improved. Indeed, the number of non-IEA annotations is continuously increasing (Figure 3). However, the rate of increase for non-IEA annotations is very slow (approximately 2,000 genes annotated in 7 years; Figure 3).

Manual curation of the entire genome is expected to take a very long time (∼13–25 years) [55]. In order to exponentially increase coverage, resolution, and accuracy of annotations, we believe that the entire research community must participate in the curation process. One approach to facilitate participation of a large number of researchers is to adopt a standard annotation format similar to Minimum Information About a Microarray Experiment (MIAME) [56]. The majority of journals now require that data from DNA microarray experiments and other high-throughput experiments be deposited in MIAME-compliant format prior to publication. Since its introduction in 2002, the Gene Expression Omnibus (GEO) [57] database at NCBI has collected 637,643 samples from 25,783 experiments on 9,385 platforms (data current as of October 2011). Similarly, it may also be beneficial to require deposition of functional study data in standard format in public repositories. A format for functional annotation can be designed or adopted from the existing formats (e.g., BioPAX, SBML). Such a format can allow researchers to specify an experimentally confirmed role of a specific transcript or a SNP in a pathway along with experimental and biological conditions. Such a repository would improve the state of functional annotations in public domains, and also enable development of the next generation of large-scale pathway analysis tools.

#### Missing condition- and cell-specific information

Most pathway knowledge bases are built by curating experiments performed in different cell types at different time points under different conditions. However, these details are typically not available in the knowledge bases. One effect of this omission is that multiple independent genes are annotated to participate in the same interaction in a pathway. This effect is so widespread that many pathway knowledge bases represent a set of distinct genes as a single node in a pathway, and is part of the standard BioPAX format. An example of this problem is the *Wnt/beta-catenin pathway* in STKE (http://stke.sciencemag.org/cgi/cm/stkecmCMC_6032; free registration is required to view this website), where the node labeled “*Genes*” represents 19 genes directly targeted by *Wnt* in different organisms (*Xenopus* [58] and human [59]) in different cells and tissues (colon carcinoma cells [60] and epithelial cells [61]). These non-specific genes introduce bias for these pathways in all existing analysis approaches. For instance, any ORA method will assign higher significance (typically an order of magnitude lower *p*-value) to a pathway with more genes. Similarly, more genes in a pathway also increase the probability of a higher pathway-level statistic in FCS approaches, yielding higher significance for a given pathway.

However, this contextual information is typically not available from most of the existing knowledge bases. A standard functional annotation format discussed above would make this information available to curators and developers. For instance, the recently proposed Biological Connection Markup Language (BCML) allows pathway representation to specify the cell or organism in which each pathway interaction occurs [62]. Furthermore, BCML can generate cell-, condition-, or organism-specific pathways based on user-defined query criteria, which in turn can be used for targeted analysis.

Existing knowledge bases do not describe the effects of an abnormal condition on a pathway (Figure 2). For example, it is not clear how the Alzheimer's disease pathway in KEGG differs from a normal pathway (http://www.genome.jp/kegg/pathway/hsa/hsa05010.html), nor it is clear which set of interactions leads to Alzheimer's disease. We are now beginning to understand that context plays an important role in pathway interactions. Information about how cell and tissue type, age, and environmental exposures affect pathway interactions will add complexity that is currently lacking.

### Methodological Challenges

#### Benchmark data sets for comparing different methods

Although multivariate pathway-level statistics outperform univariate statistics on simulated data, univariate statistics are equal to or better than multivariate statistics on real biological data [1]. This fact raises a question of how to assess performance of pathway analysis methods. One way to address the question is to compare different methods against a set of benchmark data sets.

Using simulated data [1], [18] as a benchmark has the advantage of comparing sensitivity and specificity of different methods. However, biology is more complicated than simulated data. Biological data are often affected by confounding factors such as absence of a pure division into classes, presence of outliers, experimental or technical “hidden” factors, etc. Therefore, it is desirable to use real biological data as benchmark data sets.

A number of well-studied biological data sets can be used for this purpose [21], [29], [63]–[65]. However, when using real biological data, the actual biology is never fully known. Furthermore, different definitions of the same pathway in different knowledge bases can affect performance assessment in terms of power, and the number of true positives and true negatives. For instance, GO defines different pathways for apoptosis in different cells (e.g., cardiac muscle cell apoptosis, B cell apoptosis, T cell apoptosis). It further distinguishes between induction and regulation of apoptosis. Alternatively, KEGG defines a single signaling pathway for apoptosis, and does not distinguish between induction and regulation. Hence, an approach using KEGG would identify a single pathway as significant, whereas GO could identify multiple pathways, and/or specific aspects of a single apoptosis pathway.

#### Inability to model and analyze dynamic response

While information missing from pathway knowledge bases limits analysis from a systems biology perspective, no existing approach can collectively model and analyze high-throughput data as a single dynamic system. Current approaches are designed to analyze a snapshot of a biological system by assuming that each pathway is independent of the others at a given time. A typical approach for analyzing dynamic response at the pathway level is to measure expression changes at multiple time points, and analyze each time point individually to see which pathways are significant at each time point [66], [67]. These approaches implicitly also assume that pathways at different time points are independent of each other. The lack of a model that accounts for dependence among pathways at different time points limits our ability to observe changes at a pathway level in a biological system.

For example, existing approaches for pathway analysis of gene expression profiles obtained from transplanted organ biopsies on day 1 would identify *antigen processing and presentation pathway* as significant, but probably fail to identify other downstream pathways, such as *cytokine-cytokine receptor signaling* and *T cell receptor signaling*. This failure is due to the fact that existing approaches do not account for inter-pathway dependence, such as activation of *antigen processing and presentation pathway* leading to activation of other immune pathways. The lack of methods that analyze pathways as a dynamic system is, in part, due to limitations of current molecular measurement technologies. These technologies can only quantify a snapshot of a biological system because (i) they are unable to determine protein states in a high-throughput fashion or are severely restricted in this regard; and (ii) they are unable to detect signals that propagate without affecting gene expression.

Topology-based analysis approaches can potentially model and analyze dynamic responses. For example, IF analysis models each pathway as a linear system and propagates changes in gene expression as perturbations in the system via interactions between gene products [38]–[40], [43]. However, these approaches also assume that the expression levels of all genes, measured at a specific time point, are constant and never change. This assumption almost never holds, as there are positive and negative feedback loops in pathways that continuously regulate expression of different genes. Furthermore, the assumptions made to propagate signals through the biological system and estimate expression changes of the other genes/proteins on each pathway are very gross, although they have been shown to provide useful insights.

#### Inability to model effects of an external stimuli

Gene set–based approaches often only consider genes and their products, and completely ignore the effects of other molecules participating in a pathway, such as the rate limiting step of a multi-step pathway. For instance, the amount/strength of Ca^2+^ causes different transcription factors to be activated [68], [69]. However, this information is usually not available, due to lack of experimental data, although efforts are being made to make these types of data available in the public domain [70]. None of the existing approaches fully incorporate this information in their models, although PT-based analysis methods potentially have the ability to consider some of them.

## Conclusion

In the last decade, pathway analysis has become the first choice for extracting and explaining the underlying biology for high-throughput molecular measurements. Today, virtually every bioinformatics study looks for statistically significant pathways as either biological interpretation or validation of computationally derived results. This paper discusses the evolution of pathway analysis methods of high-throughput molecular measurements in the last decade, distinctly divided into three generations based on the type of analysis they performed. Although widely adopted, the first generation of pathway analysis methods, ORA methods, decouple molecular measurements from functional analysis and assume that genes and pathways are independent of each other. The second-generation FCS methods address these limitations. PT-based methods further improve FCS methods by considering the number and type of interactions between genes, which FCS methods ignore.

However, despite these efforts, there are outstanding annotation and methodological challenges. First, low resolution knowledge bases, missing condition- and cell-specific information, and incomplete annotations restrict development of the next-generation pathway analysis methods. Second, the inability to integrate the dynamic nature of a biological system in analysis limits the utility of existing methods. However, despite these hurdles, as the number and type of functional annotations increase, coupled with technological advances and analysis methods that provide better guidance for strategic planning for subsequent biological experiments, the utility of pathway analysis and confidence in results will likely improve. The community must address these challenges collectively to move pathway analysis into the next generation that is able to utilize the new high-throughput technologies in order to better understand large biological systems and to increase the specificity, sensitivity, and relevance of pathway analysis, and consequently, its utility.

## Supporting Information

Text S1 Description of the linear model used by IF analysis.
(PDF)

Text S2 Feature comparison of a few existing pathway analysis tools in each generation.
(PDF)

Table S1 Comparison of 11 ORA pathway analysis tools and analysis features available in them.
(PDF)

Table S2 Comparison of seven FCS pathway analysis tools and analysis features available in them.
(PDF)

Table S3 Comparison of three PT-based pathway analysis tools and analysis features available in them.
(PDF)

Table S4 NCBI Entrez Gene statistics for the types of genes annotated for humans.
(PDF)
