# Supplementary material for: Ten Years of Pathway Analysis: Current Approaches and Outstanding Challenges
Source: PLoS Comput Biol. 2012 Feb 23;8(2):e1002375. doi: 10.1371/journal.pcbi.1002375 (PMC3285573; doi:10.1371/journal.pcbi.1002375)
Supplement: Text S1 — Description of the linear model used by IF analysis. (PDF) [file pcbi.1002375.s001.pdf]

10 Years of Pathway Analysis: Current Approaches and Outstanding Challenges - Supplementary Notes

Purvesh Khatri[1,2,*], Marina Sirota[1,2], Atul J Butte[1,2,*]
1 Division of Systems Medicine, Department of Pediatrics, Stanford University School of Medicine, Stanford, CA 94305
2 Lucile Packard Children's Hospital, 725 Welch Road, Palo Alto, CA 94304
∗ E-mail: pkhatri@stanford.edu, abutte@stanford.edu

S1 Impact Factor Analysis

Impact Factor (IF) analysis [1, 2] combines both ORA and FCS approach, while accounting for the topology of the pathway. IF analysis computes Perturbation Factor (PF) for each gene in each pathway, which is a gene-level statistic, as follows:

$$PF(g_i) = \Delta F(g_i) + \sum_{j=1}^{n} \beta_{ji} \cdot \frac{PF(g_j)}{N_{ds}(g_j)} \qquad (1)$$

In Eq. 1, the first term, $\Delta F(g_i)$, represents the signed normalized measured expression change (i.e., fold change) of the gene g_i. The second term in Eq. 1 accounts for the topology of the pathway, where gene g_j is upstream of gene g_i. In the second term, β_{ji} represents the type and strength of interaction between g_i and g_j. If g_j activates g_i, $\beta_{ji} = 1$, and if g_j inhibits g_i, $\beta_{ji} = -1$. Note that the PF of the upstream gene g_j is normalized by the number of downstream genes it interacts with, $N_{ds}(g_i)$. The second term is repeated for every gene g_j that is upstream of gene g_i.

After computing PF for each gene, pathway-level statistic, Impact Factor (IF), is computed using Eq. 2:

$$IF(P_i) = log\left(\frac{1}{p_i}\right) + \frac{\left|\sum_{g \in P_i} PF(g)\right|}{N_{de}(P_i)} \qquad (2)$$

In Eq. 2, the first term captures the significance of the given pathway P_i as provided by ORA, where p_i corresponds to the probability of obtaining a value of the statistic used at least as extreme as the one observed when the null hypothesis is true. Because IF should be large for severely impacted pathways (i.e., small p-values), the first term uses $1/p_i$ rather than p_i. The log function is necessary to map the exponential scale of the p-values to a linear scale in order to keep the model linear. The second term sums up the values of the PFs for all genes g on the given pathway P_i, and is normalized by the number of differentially expressed genes on the given pathway P_i.

Note that Eq. 1 essentially describes the perturbation factor PF for a gene g_i as a linear function of the perturbation factors of all genes in a given pathway. Therefore, the set of all equations defining the PFs for all genes in a given pathway P_i form a system of simultaneous equations. Expanding and re-arranging Equation 1 for all genes $g_1, g_2, \ldots, g_n$ in a pathway P_i can be re-written as follows:

$$\begin{pmatrix} PF(g_1) \\ PF(g_2) \\ \cdots \\ PF(g_n) \end{pmatrix} = \begin{pmatrix} 1 - \frac{\beta_{11}}{N_{ds(g_1)}} & -\frac{\beta_{21}}{N_{ds(g_2)}} & \cdots & -\frac{\beta_{n1}}{N_{ds(g_n)}} \\ -\frac{\beta_{12}}{N_{ds(g_1)}} & 1 - \frac{\beta_{22}}{N_{ds(g_2)}} & \cdots & -\frac{\beta_{n2}}{N_{ds(g_n)}} \\ \cdots & \cdots & \cdots & \cdots \\ -\frac{\beta_{1n}}{N_{ds(g_1)}} & -\frac{\beta_{2n}}{N_{ds(g_2)}} & \cdots & 1 - \frac{\beta_{nn}}{N_{ds(g_n)}} \end{pmatrix}^{-1} \begin{pmatrix} \alpha(g_1) \cdot \Delta E(g_1) \\ \alpha(g_2) \cdot \Delta E(g_2) \\ \cdots \\ \alpha(g_n) \cdot \Delta E(g_n) \end{pmatrix}$$

After computing the PFs of all genes in a given pathway as the solution of this linear system, Eq. 2 is used to calculate the impact factor of each pathway. The impact factor of each pathway is then used as

a score to assess the impact of a given gene expression data set on all pathways (the higher the impact factor the more significant the pathway).

References

1. Draghici S, Khatri P, Tarca AL, Amin K, Done A, et al. (2007) A systems biology approach for pathway level analysis. Genome Research 17: 1537–1545.

2. Khatri P, Drăghici S, Tarca AL, Hassan SS, Romero R (2007) A system biology approach for the steady-state analysis of gene signaling networks. In: 12th Iberoamerican Congress on Pattern Recognition, CIARP 2007. Valparaiso, Chile.
