# Supplementary material for: Ten Years of Pathway Analysis: Current Approaches and Outstanding Challenges
Source: PLoS Comput Biol. 2012 Feb 23;8(2):e1002375. doi: 10.1371/journal.pcbi.1002375 (PMC3285573; doi:10.1371/journal.pcbi.1002375)
Supplement: Text S2 — Feature comparison of a few existing pathway analysis tools in each generation. (PDF) [file pcbi.1002375.s002.pdf]

10 Years of Pathway Analysis: Current Approaches and Outstanding Challenges - Supplementary Notes

Purvesh Khatri[1,2,*], Marina Sirota[1,2], Atul J Butte[1,2,*]
1 Division of Systems Medicine, Department of Pediatrics, Stanford University School of Medicine, Stanford, CA 94305
2 Lucile Packard Children's Hospital, 725 Welch Road, Palo Alto, CA 94304
∗ E-mail: pkhatri@stanford.edu, abutte@stanford.edu

S2 Feature comparison of the existing pathway analysis tools

Supplementary tables 1, 2 and 3 list some of the available tools for pathway analysis of high-throughput data separated by class of method. Furthermore, commercial tools are arbitrarily excluded from the comparison. We have also arbitrarily excluded some of the tools that provide identical functionalities.

S2.1 Gene-level Statistic

As shown in Tables 2 and 3, a large number of gene-level statistics have been proposed for ranking individual genes before computing gene set statistic in FCS methods. Although the choice of a gene-level statistic has been found to have negligible effect on identifying significantly enriched gene sets in simulated data and real biological data [1], one should keep in mind that when the number of biological replicates are few, a regularized statistic may be better. Furthermore, untransformed gene-level statistics failed to identify pathways with both up- and down-regulated genes, in which case, transformation of gene-level statistics (e.g., absolute values, squared values, ranks, etc.) is preferable [1, 2]. Another example of gene-level statistics is using a linear model for obtaining two sample t-statistic instead of conventional t-statistic [2]. When the sample size is large, a linear model can be complex to allow inclusion of more variables and interactions [2].

S2.2 Gene Set Statistic

A gene set statistic can be multivariate [3–7] and account for interdependencies among genes, or univariate [2, 8] and disregard interdependencies among genes. Irrespective of its type, the power of a statistic can depend on the proportion of differentially expressed genes in a pathway, size of the pathway and amount of correlation between genes in a pathway. On simulated data, Glazko *et al.* showed that irrespective of the size of pathways and proportion of the differentially expressed genes in pathways, power of univariate and multivariate test is most affected by correlations between genes in pathways, and is inversely proportional to amount of correlation between genes in pathways [9]. Although at low correlations between genes, all tests had similar power, at higher correlations, N-statistic (multivariate statistic) had more power than other multivariate (Hotelling's T^2, Dempster's T_1) and univariate (average absolute t-statistic, average squared t-statistic) statistics. Hotelling's T^2 test is expected to have low power when correlation between genes in a pathway is high as Hotelling's test is specifically designed to penalize correlation [1]. Furthermore, when correlations between genes are kept constant, power of these tests was proportional to the size of pathways and proportions of genes differentially expressed on the pathways with multivariate statistics performing better than univariate statistics [9].

Despite demonstrated higher power of multivariate statistics in simulated data, when applied to real biological data, univariate statistics showed more power at stringent cutoffs (P-value ≤ 0.001), and had equal power as multivariate statistics at less stringent cutoffs (P-value ≤ 0.05) [9]. Furthermore, because different statistics test different hypotheses (e.g., N-statistic tests equality of two multivariate distributions, where as average absolute t-statistic tests equality of means of two distributions), each statistic identified a set of pathways that were not identified by the other statistics. The pathways

uniquely identified by each statistics were true positives, suggesting that the number of false negatives may be high for commonly used statistics [9]. Interestingly, only Hotelling's T^2 was able to identify truly differentially regulated pathways when the proportions of differentially expressed genes in the pathways were low. On the other hand, a comparison of six gene set statistics, which did not include Hotelling's T^2, on simulated data found that each of them had difficulty in identifying those gene sets where only a subset of genes were differentially expressed [1]. Taken together these results suggest that Hotelling's T^2 has higher sensitivity when a small number of genes are differentially expressed in a pathway. Tables 2 and 3 lists the options available in the existing tools for gene set statistics.

S2.3 Assessing Statistical Significance of Pathways

The interpretation of the statistical significance of a pathway depends on the null hypothesis being tested, which in turn is closely tied to sampling method employed [1, 8, 10]. The null hypotheses tested by the current pathway analysis approaches can be broadly divided into two categories: i) competitive and ii) self-contained null hypothesis. A competitive null hypothesis states that *the genes in a given pathway are at most as often differentially expressed as the genes not in the pathway*, whereas a self-contained hypothesis states that *no genes in a given pathways are differentially expressed* [1, 8, 10, 11]. In other words, a competitive null hypothesis compares a set of genes in a given pathway with a set of genes that are not in the pathway, whereas a self-contained null hypothesis ignores the genes that are not in the pathway. Self-contained null hypotheses reject more null hypotheses (i.e., have more power) than competitive null hypotheses as they are more restrictive [8, 10]. Though desirable, the higher power of a self-contained null hypothesis can be problematic when there is a large number of differentially expressed genes, as almost all pathways may be called significant. Furthermore, a self-contained hypothesis test is a generalization of a single gene testing method by treating a gene set with single gene same as a gene set with more than one gene, whereas, a competitive hypothesis treats a gene set with single gene very differently from a gene set with more than one genes [10].

Another difference between the two hypotheses is their sampling units. A "sampling unit" refers to the entity that will be used to repeat an experiment. A competitive null hypothesis samples genes, whereas a self-contained null hypothesis samples subjects [8, 10]. A typical experiment measures the same genes in a set of subjects. When repeating the experiment to reproduce the results, one would measure the same set of genes in another set of subjects. Hence, in a typical experiment sampling units are subjects. Consequently, using genes as sampling units turns a typical experiment design on its head. Therefore, despite continued widespread use of gene sampling approaches in practice, a strong consensus has started to form in the literature in favor of subject sampling (i.e., self-contained null) [1, 2, 8–10].

One of the most important differences between competitive and self-contained null hypothesis is the inclusion of correlation structure in null hypothesis model. By randomly selecting genes for a given pathway, competitive null disrupts the correlation structure between genes, and hence, ignores the correlation structure between genes in null model. On the other hand, because self-contained null permutes subjects and ignores the genes not in a given pathway, it includes the correlation structure between genes in null model. One could argue that if the presence of correlation in measurements is not due to purely technical or experimental reasons (e.g., unspecific hybridization or probe redundancy in case of microarrays), it may be regarded as a biologically meaningful signal, which therefore should not be incorporated in the random model for the null hypothesis. This argument is fundamentally flawed. We note that in any high throughput experiment, not all molecules are expected to be different. For instance, when comparing a cancer sample with a normal sample, many pathways (e.g., cell division) are not expected to be affected. Consequently, the genes involved in these pathways will have some correlation between them, although they may not be differentially expressed. In other words, there will always be some correlation between genes and proteins in high throughput experiment data, and therefore, should be incorporated in the random model. Inclusion of correlation structure in random model is another reason in favor of self-contained null hypothesis as an appropriate test to identify significant pathways.

Virtually all existing ORA approaches use 2 x 2 table methods [12] (Table 1), which test competitive null hypothesis by comparing the proportion of differentially expressed genes in a pathway with the proportion of differentially expressed genes not in the pathway. Because the input for the majority of the existing ORA-based tools is a list of significant genes, without any expression data associated with the list, there is no opportunity for subject sampling. On the other hand, FCS-based tools make use of all data available from an experiment, which in turn provides opportunity for either subject or gene sampling. All available FCS methods listed in Table 2 allow subject sampling (i.e., phenotype permutation).

S2.4 Correction for Multiple Hypotheses

Despite the generally accepted importance of correction for multiple hypotheses (see `http://www.silicongenetics.com/Support/GeneSpring/GSnotes/analysis_guides/mtc.pdf` to understand the need for the correction), several ORA tools do not provide correction for multiple hypotheses, including GoMiner, FatiGO, GenMAPP/MAPPFinder, and GOTM [12]. Tables 1, 2, and 3 list the choices available in existing tools for multiple hypotheses correction. Among the available choices, Bonferroni, Sidak and Holm's corrections are very conservative, and not suitable when the number of pathways considered is large.

Recently, false discovery rate (FDR) is considered to be more appropriate. Because underlying distributions of data are generally unknown, several variations of FDR with different assumptions have been proposed in literature, which include Benjamini-Hochberg (BH) correction [13], Benjamini-Yekutieli (BY) correction [14], positive FDR (pFDR) [15,16], adaptive Benjamini-Hochberg (ABH) [17], significant analysis of microarrays (SAM) [18], Storey's q-value [15], and re-sampling based approaches [19–22]. Presence of dependence in molecular measurements has been shown to affect power of FDR significantly [23]. Using random correlation matrices, Kim *et al.* compared seven different FDR methods by varying the number of correlated genes and the strength of correlation [24]. Their results show that in the presence of correlation BY is the most conservative FDR, whereas SAM and q-value overestimate FDR by 3 to 6%. BH and its variants (ABH, re-sampling based approaches) consistently underestimate FDR in the presence of dependence. Furthermore, the strength of correlation between genes also has significant effect on FDR [24].

References

1. Ackermann M, Strimmer K (2009) A general modular framework for gene set enrichment analysis. BMC Bioinformatics 10: 47.

2. Jiang Z, Gentleman R (2007) Extensions to gene set enrichment. Bioinformatics 23: 306–13.

3. Kong SW, Pu WT, Park PJ (2006) A multivariate approach for integrating genome-wide expression data and biological knowledge. Bioinformatics 22: 2373–80.

4. Lu Y, Liu PY, Xiao P, Deng HW (2005) Hotelling's T2 multivariate profiling for detecting differential expression in microarrays. Bioinformatics 21: 3105-13.

5. Xiong H (2006) Non-linear tests for identifying differentially expressed genes or genetic networks. Bioinformatics 22: 919-923.

6. Hummel M, Meister R, Mansmann U (2008) GlobalANCOVA: exploration and assessment of gene group effects. Bioinformatics 24: 78-85.

7. Klebanov L, Glazko G, Salzman P, Yakovlev A, Xiao Y (2007) A multivariate extension of the gene set enrichment analysis. J Bioinform Comput Biol 5: 1139-53.

8. Tian L, Greenberg SA, Kong SW, Altschuler J, Kohane IS, et al. (2005) Discovering statistically significant pathways in expression profiling studies. Proc Natl Acad Sci U S A 102: 13544–9.

9. Glazko G, Emmert-Streib F (2009) Unite and conquer: univariate and multivariate approaches for finding differentially expressed gene sets. Bioinformatics 25: 2348-2354.

10. Goeman JJ, Buhlmann P (2007) Analyzing gene expression data in terms of gene sets: methodological issues. Bioinformatics 23: 980–7.

11. Efron B, Tibshirani R (2007) On testing the significance of sets of genes. Annals of Applied Statistics 1: 107-129.

12. Khatri P, Draghici S (2005) Ontological analysis of gene expression data: current tools, limitations, and open problems. Bioinformatics 21: 3587–3595.

13. Benjamini Y, Hochberg Y (1995) Controlling the false discovery rate: A practical and powerful approach to multiple testing. Journal of The Royal Statistical Society B 57: 289–300.

14. Benjamini Y, Yekutieli D (2001) The control of the false discovery rate in multiple testing under dependency. Annals of Statistics 29: 1165–1188.

15. Storey JD (2002) A direct approach to false discovery rates. Journal of the Royal Statistical Society, Series B (Statistical Methodology) 64: 479–498.

16. Storey J (2003) The positive false discovery rate: a Bayesian interpretation and the q-value. Annals of Statistics 31: 2013-2035.

17. Benjamini Y, Krieger AM, Yekutieli D (2006) Adaptive linear step-up procedures that control the false discovery rate. Biometrika 93: 491-507.

18. Tusher VG, Tibshirani R, Chu G (2001) Significance analysis of microarrays applied to the ionizing radiation response. Proc Natl Acad Sci 98: 5116–5121.

19. Yekutieli D, Benjamini Y (1999) Resampling-based false discovery rate controlling multiple test procedures for correlated test statistics. Journal of Statistical Planning and Inference 82: 171-196.

20. Westfall PH, Young SS (1993) Resampling-based multiple testing: Examples and Methods for p-value adjustment. New York: Wiley.

21. Black MA (2004) A note on the adaptive control of false discovery rates. Journal of Royal Statistical Society 66: 297-304.

22. Langaas M, Lindqvist BH (2005) Estimating the proportion of true null hypotheses, with application to DNA microarray data. Journal of Royal Statistical Society 67: 555-572.

23. Efron B (2007) Correlation and Large-Scale Simultaneous Significance Testing. Journal of the American Statistical Association 102: 93–103.

24. Kim KI, van de Wiel MA (2008) Effects of dependence in high-dimensional multiple testing problems. BMC Bioinformatics 9: 114.
