# Supplementary material for: Ten Years of Pathway Analysis: Current Approaches and Outstanding Challenges
Source: PLoS Comput Biol. 2012 Feb 23;8(2):e1002375. doi: 10.1371/journal.pcbi.1002375 (PMC3285573; doi:10.1371/journal.pcbi.1002375)
Supplement: Table S1 — Comparison of 11 ORA pathway analysis tools and analysis features available in them. (PDF) [file pcbi.1002375.s003.pdf]

10 Years of Pathway Analysis: Current Approaches and Outstanding Challenges - Supplementary Notes

Purvesh Khatri[1,2,*], Marina Sirota[1,2], Atul J Butte[1,2,*]
1 Division of Systems Medicine, Department of Pediatrics, Stanford University School of Medicine, Stanford, CA 94305
2 Lucile Packard Children's Hospital, 725 Welch Road, Palo Alto, CA 94304
∗ E-mail: pkhatri@stanford.edu, abutte@stanford.edu

Table S1. ORA pathway analysis tools.

Name	Scope of Analysis	P-value	Correction for Multiple Hypotheses	Availability
Onto-Express	GO	Hypergeometric, binomial, chi-square	FDR, Bonferroni, Sidak, Holm	Web
GenMAPP/ MAPPFinder	GO, KEGG, MAPP	Percentage/z-score	None	Standalone
(High throughput) GoMiner	GO	Relative enrichment, Hypergeometric	None	Standalone, Web
FatiGO	GO, KEGG	Hypergeometric	None	Web
GOstat	GO	Chi-square	FDR	
GOTree Machine	GO	Hypergeometric	None	Web
FuncAssociate	GO	Hypergeometric	Bootstrap	Web
GOToolBox	GO	Hypergeometric	Bonferroni, Holm, FDR, Hommel, Hochberg	
GeneMerge	GO	Hypergeometric	Bonferroni	Web
GOEAST	GO	Hypergeometric, Chi-square	Benjamini-Yekutieli	Web
ClueGO	GO, KEGG, BioCarta, User defined	Hypergeometric	Bonferroni, Bonferroni step-down, Benjamini-Hochberg	Standalone
