# Supplementary material for: Ten Years of Pathway Analysis: Current Approaches and Outstanding Challenges
Source: PLoS Comput Biol. 2012 Feb 23;8(2):e1002375. doi: 10.1371/journal.pcbi.1002375 (PMC3285573; doi:10.1371/journal.pcbi.1002375)
Supplement: Table S2 — Comparison of seven FCS pathway analysis tools and analysis features available in them. (PDF) [file pcbi.1002375.s004.pdf]

10 Years of Pathway Analysis: Current Approaches and Outstanding Challenges - Supplementary Notes

Purvesh Khatri[1,2,*], Marina Sirota[1,2], Atul J Butte[1,2,*]
1 Division of Systems Medicine, Department of Pediatrics, Stanford University School of Medicine, Stanford, CA 94305
2 Lucile Packard Children's Hospital, 725 Welch Road, Palo Alto, CA 94304
∗ E-mail: pkhatri@stanford.edu, abutte@stanford.edu

Table S2. FCS pathway analysis tools.

Name	Scope of Analysis	Gene-level Statistic	Gene Set Statistic	P-value	Correction for Multiple Hypotheses	Availability
GSEA	GO, KEGG, BioCarta, MAPP, transcription factors, microRNA, cancer molecules	Signal-to-noise ratio, t-test, cosine, euclidian and manhattan distance, Pearson correlation, (log2) fold-change, log difference	Kolmogorov-Smirnov	Phenotype permutation, Gene set permutation	FDR	Standalone, R package
sigPathway	GO, KEGG, BioCarta, humanpaths	t-statistic	Wilcoxon rank sum	Phenotype permutation, Gene set permutation	FDR (NPMLE)	R package
Category	GO, KEGG	t-statistic		Phenotype permutation	NA	R package
SAFE	GO, KEGG, PFAM	Student's t-test, Welch's t-test, SAM t-test, f-statistic, Cox proportional hazards model, linear regression	Wilcoxon rank sum, Fisher's exact test statistic, Pearson's test, t-test of average difference	Phenotype permutation	FWER (Bonferroni, Holm's step-up), FDR (Benjamini-Hochberg, Yekutieli-Benjamini)	R package
GlobalTest	GO, KEGG	NA	simple and multinomial logistic regression, Q-statistics mean	Phenotype permutation, asymptotic distribution, Gamma distribution	NA	R package
PCOT2	User specified	Hotelling's T^2		Phenotype permutation, gene set permutation	FDR (Benjamini-Hochberg, Yekutieli-Benjamini), FWER (Bonferroni, Holm, Hochberg, Hommel)	R package
SAM-GS	User specified	d-statistic	sum of squared d-statistic	Phenotype permutation	FDR	Excel plug-in
