# Supplementary material for: Ten Years of Pathway Analysis: Current Approaches and Outstanding Challenges
Source: PLoS Comput Biol. 2012 Feb 23;8(2):e1002375. doi: 10.1371/journal.pcbi.1002375 (PMC3285573; doi:10.1371/journal.pcbi.1002375)
Supplement: Table S3 — Comparison of three PT-based pathway analysis tools and analysis features available in them. (PDF) [file pcbi.1002375.s005.pdf]

10 Years of Pathway Analysis: Current Approaches and Outstanding Challenges - Supplementary Notes

Purvesh Khatri[1,2,*], Marina Sirota[1,2], Atul J Butte[1,2,*]
1 Division of Systems Medicine, Department of Pediatrics, Stanford University School of Medicine, Stanford, CA 94305
2 Lucile Packard Children's Hospital, 725 Welch Road, Palo Alto, CA 94304
∗ E-mail: pkhatri@stanford.edu, abutte@stanford.edu

Table S3. PT-based pathway analysis tools.

Name	Scope of Analysis	Gene-level statistic	P-value	Correction for Multiple Hypotheses	Availability
ScorePAGE	KEGG (metabolic)	(correlation, covariance, cosine, dot product) + Number of reactions	Gene set permutation	FDR (Benjamini-Hochberg)	NA
Pathway-Express/SPIA	KEGG (signaling)	Number and type of interactions, fold-change	hypergeometric, binomial	FDR	R package, web
