# Supplementary material for: Ten Years of Pathway Analysis: Current Approaches and Outstanding Challenges
Source: PLoS Comput Biol. 2012 Feb 23;8(2):e1002375. doi: 10.1371/journal.pcbi.1002375 (PMC3285573; doi:10.1371/journal.pcbi.1002375)
Supplement: Table S4 — NCBI Entrez Gene statistics for the types of genes annotated for humans. (PDF) [file pcbi.1002375.s006.pdf]

10 Years of Pathway Analysis: Current Approaches and Outstanding Challenges - Supplementary Notes

Purvesh Khatri[1,2,*], Marina Sirota[1,2], Atul J Butte[1,2,*]
1 Division of Systems Medicine, Department of Pediatrics, Stanford University School of Medicine, Stanford, CA 94305
2 Lucile Packard Children's Hospital, 725 Welch Road, Palo Alto, CA 94304
∗ E-mail: pkhatri@stanford.edu, abutte@stanford.edu

Table S4. NCBI Entrez Gene statistics for the types of genes annotated for humans.

**Type**	**Number of genes**
Protein-coding	21,574
Pseudogenes	14,162
miscRNA	4,818
tRNA	598
snoRNA	370
snRNA	78
rRNA	24
scRNA	5
ncRNA	1
Other	829
Unknown	2,824
**Total**	**45,283**
